# Uncovering the Hidden Connections Between PCOS and Alzheimer’s Disease: A Two-Sample Mendelian Randomization Perspective

**DOI:** 10.5812/ijem-159124

**Published:** 2025-04-30

**Authors:** Farzaneh Sadat Motafeghi, Mahdi Akbarzadeh, Samaneh Talebi, Danial Habibi, Sahand Tehrani Fateh, Hamid Alavi Majd, Mehdi Hedayati, Fereidoun Azizi, Maryam Sadat Daneshpour, Fahimeh Ramezani Tehrani

**Affiliations:** 1 Reproductive Endocrinology Research Center, Research Institute for Endocrine Molecular Biology, Research Institute for Endocrine Sciences, Shahid Beheshti University of Medical Sciences, Tehran, Iran; 2 Cellular and Molecular Endocrine Research Center, Research Institute for Endocrine Molecular Biology, Research Institute for Endocrine Sciences, Shahid Beheshti University of Medical Sciences, Tehran, Iran; 3 Department of Biostatistics, School of Allied Medical Sciences, Shahid Beheshti University of Medical Sciences, Tehran, Iran; 4 Social Determinants of Health Research Center, Health Research Institute, Babol University of Medical Sciences, Babol, Iran; 5 School of Medicine, Tehran University of Medical Sciences, Tehran, Iran; 6 Endocrine Research Center, Research Institute for Endocrine Disorders, Research Institute for Endocrine Sciences, Shahid Beheshti University of Medical Sciences, Tehran, Iran; 7 Foundation for Research and Education Excellence, Vestavia Hills, AL, USA

**Keywords:** Polycystic Ovary Syndrome, Alzheimer Disease, Mendelian Randomization Analysis, Genetic Epidemiology, Causality

## Abstract

**Background:**

Polycystic ovary syndrome (PCOS) and Alzheimer’s disease (AD) are two prevalent and complex conditions characterized by overlapping features such as metabolic dysfunction, hormonal imbalance, and chronic inflammation. These commonalities raise the possibility of a shared causal pathway. However, observational studies often face limitations due to confounding factors, complicating causal inference.

**Objectives:**

The present study aimed to explore the causal link between PCOS and AD through Mendelian randomization (MR) analysis.

**Methods:**

We conducted a two-sample MR analysis using summary-level data from two large genome-wide association studies (GWAS). For the exposure, genetic variants strongly associated with PCOS were obtained from a GWAS meta-analysis involving 10,074 cases and 103,164 controls of European ancestry. For the outcome, AD data were sourced from a separate GWAS comprising 1,036,225 cases and 90,338 controls, also of European descent. Multiple MR approaches were employed, with inverse variance weighted (IVW) as the primary method, supported by MR-Egger, weighted median, and weighted mode methods. Sensitivity analyses were performed to assess the robustness of the findings.

**Results:**

The two-sample MR analysis did not provide evidence for a significant causal effect of genetically predicted PCOS on AD risk. The initial IVW analysis using all instrumental variables (IVs) yielded an odds ratio (OR) of 0.967 [95% confidence interval (CI): 0.905 - 1.03; P = 0.311]. After removing outlier single nucleotide polymorphisms (SNPs) based on sensitivity analyses, the refined IVW model showed an OR of 0.93 (95% CI: 0.866 - 1.002; P = 0.057), indicating no statistically significant association. The results were consistent across various MR methods, and sensitivity tests confirmed the robustness of the findings.

**Conclusions:**

This MR study found no evidence of a significant causal relationship between genetically predicted PCOS and AD. These findings suggest that genetic predisposition to PCOS does not increase the risk of AD, indicating that previously observed associations in epidemiological studies may not reflect a causal link. Further studies are needed to explore alternative explanations beyond genetic causality.

## 1. Background

Polycystic ovary syndrome (PCOS) is a prevalent endocrine disorder affecting 4 - 21% of women in their reproductive years globally. It is characterized by symptoms such as irregular menstruation, elevated androgen levels, and impaired ovulation (1-3). Beyond reproductive challenges, PCOS is associated with significant metabolic disturbances, including insulin resistance, obesity, and chronic inflammation, which can have profound systemic consequences. These metabolic derangements, along with hormonal imbalances, have raised concerns about potential links between PCOS and neurodegenerative diseases, particularly Alzheimer’s disease (AD) (4, 5).

Alzheimer’s disease is a neurodegenerative condition defined by a gradual decline in cognitive abilities, memory impairment, and the buildup of amyloid-beta plaques and neurofibrillary tangles in the brain. Notably, both PCOS and AD share several common risk factors, including insulin resistance, chronic inflammation, and oxidative stress. Insulin resistance, a hallmark of PCOS, can impair insulin signaling in the brain, contributing to cognitive dysfunction and exacerbating amyloid-beta deposition (6, 7). Furthermore, the chronic inflammatory state associated with PCOS can contribute to neuroinflammation, further accelerating neurodegeneration.

Recent research suggests a possible association between PCOS and AD. The metabolic and hormonal imbalances in PCOS could contribute to neurodegeneration. Insulin resistance, a hallmark of PCOS, may worsen amyloid plaque buildup in AD (8, 9). Androgen excess, high luteinizing hormone (LH) relative to follicle-stimulating hormone (FSH), and low vitamin D levels in PCOS might contribute to neuroinflammation and neuronal loss, processes also seen in AD (7).

To further elucidate the causal relationship between PCOS and AD, robust research methodologies are needed. Mendelian randomization (MR) is an analytical approach employed to investigate potential causal relationships between two unrelated conditions. By harnessing the principle of random allocation of genetic variants (alleles) during gamete formation, MR studies typically utilize data from unrelated individuals, assuming that genotype and environmental factors are independent when specific covariates are considered. The application of MR in exploring the PCOS-AD connection holds promise for uncovering potential causal pathways and informing targeted interventions (10).

## 2. Objectives

The present study aimed to explore the causal link between PCOS and AD through MR analysis. By employing genetic data, we aim to enhance our understanding of the connection between metabolic-endocrine disorders and neurodegenerative diseases, potentially paving the way for future therapeutic interventions.

## 3. Methods

### 3.1. Study Design

**Figure 1.**
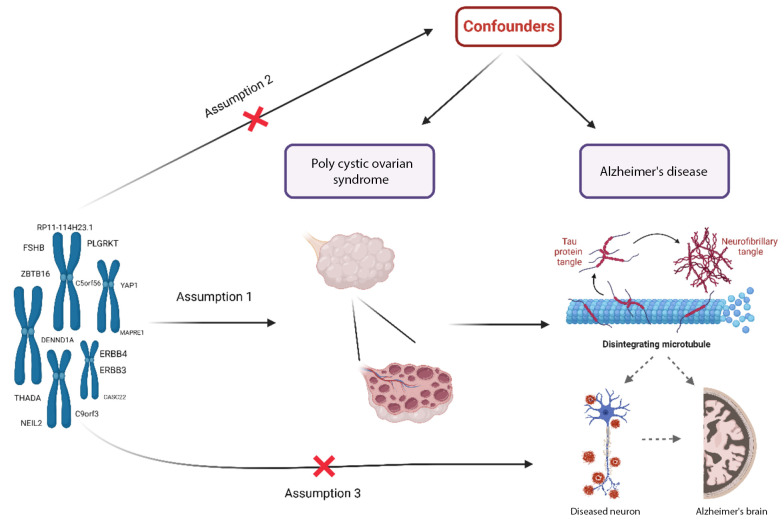
The framework of Mendelian randomization (MR) analysis relies on several critical assumptions: A, the genetic variations must have a strong association with polycystic ovary syndrome (PCOS); B, the genetic variations should not be linked to any known or unknown confounders; and C, the single nucleotide polymorphisms (SNPs) must influence the risk of AD solely through their effect on PCOS, without involvement in alternative pathways.

This two-sample MR study investigates the causal impact of PCOS on AD, following the framework illustrated in Figure 1. The MR analysis is based on three key assumptions: First, the selected single nucleotide polymorphisms (SNPs) used as instrumental variables (IVs) must have a strong association with PCOS. Second, the IVs should not be linked to any confounding factors that might affect the relationship between PCOS and AD. Finally, the influence of the IVs on AD must occur exclusively through their effect on PCOS, without any direct association with AD.

The genome-wide association study (GWAS) data for the exposure were obtained from a recent genome-wide association meta-analysis of PCOS, encompassing 10,074 cases and 103,164 controls across seven European cohorts. The diagnosis of PCOS was based on one of the following: The NIH criteria, requiring hyperandrogenism (HA) and ovulatory dysfunction (OD); the Rotterdam criteria, which mandate at least two of three features — HA, OD, or polycystic ovarian morphology (POCM); or self-reported questionnaire data (11). The GWAS data for the outcome were derived from a genome-wide association meta-analysis of AD, with 90,338 cases and 1,036,225 controls collated from 13 European cohorts (12). We obtained GWAS summary statistics of AD and PCOS from Psychiatric Genomics Consortium (PGC) and University of Cambridge Repository, respectively. Summary-level data details for each GWAS are provided in Table 1. 

**Table 1.** Summary of Genome-Wide Association Study Datasets Used in the Two-Sample Mendelian Randomization Analysis ^a^

Variables	Phenotype	Reporting Traits in the Database	Source	PMID	Sample Size	Ethnicity
**Exposure**	PCOS	PCOS	GWAS catalog	30566500	10074 (case), 103,164 (control)	European
**Outcome**	AD	AD	PGC	34493870	1,036,225 (case), 90,338 (control)	European

Abbreviations: PCOS, polycystic ovary syndrome; AD, Alzheimer’s disease; GWAS, genome-wide association studies; PGC, Psychiatric Genomics Consortium.

^a^ The table includes phenotype definitions, data sources, sample sizes for cases and controls, and population ancestry.

### 3.2. Instrumental Single Nucleotide Polymorphisms Selection

A GWAS threshold of P < 5 × 10^-8^ was used to extract significant related SNPs with PCOS. To minimize correlations between the selected SNPs, linkage disequilibrium (LD) clumping was restricted to r^2^ < 0.001 in a clumping distance of 10,000 kb window. The exposure and outcomes data were harmonized after clumping to ensure that alleles were aligned, and the presence of ambiguous and palindromic variants was investigated. In addition, SNPs with a minor allele frequency (MAF) of less than 0.01 were excluded, and the PhenoScanner results were examined to determine potential confounders. F-statistics were employed to evaluate the issue of weak IVs in MR analysis (13), calculated through = R^2^ × (N - 2)/1 - R^2^ in which R^2^ (2 × MAF × Beta^2^_exp_) and N represent the total variance of the extracted SNPs and sample size, respectively. Finally, IVs with F > 10 were included in our statistical analysis. Table 2 contains detailed information about the SNPs.

**Table 2.** Genetic Variants Single Nucleotide Polymorphisms Used as Instrumental Variables for Polycystic Ovary Syndrome in the Mendelian Randomization Analysis ^a^

SNP	Effect Allele	Other Allele	EAF	β	SE	Gene	P-Value	F Statistic
**rs11031005**	T	C	0.8537	-0.1593	0.0223	FSHB	8.664E-13	51.03
**rs11225154**	A	G	0.0941	0.1787	0.0272	YAP1	5.438E-11	43.16
**rs13164856**	T	C	0.7291	0.1235	0.0193	C5orf56	1.453E-10	40.95
**rs1784692**	T	C	0.8237	0.1438	0.0226	ZBTB16	1.876E-10	40.49
**rs1795379**	T	C	0.2398	-0.1174	0.0195	RP11-114H23.1	1.808E-09	36.25
**rs2178575**	A	G	0.1512	0.1663	0.0219	ERBB4	3.344E-14	57.66
**rs2271194**	A	G	0.4284	-0.0933	0.0168	ERBB3	2.946E-08	30.84
**rs7563201**	A	G	0.4507	-0.1081	0.0172	THADA	3.678E-10	39.49
**rs7864171**	A	C	0.3078	0.1097	0.0197	C9orf3	2.51E-08	31.01
**rs9696009**	A	G	0.0679	0.202	0.0311	DENND1A	7.958E-11	42.19

Abbreviation: SNP, Single nucleotide polymorphism.

^a^ The table includes the effect and other alleles, effect allele frequency, beta coefficients, standard errors, associated genes, P-values for association with polycystic ovarian syndrome, and F-statistics to evaluate instrument strength (F > 10 indicates strong instrument).

### 3.3. Statistical Analysis

The inverse variance weighted (IVW) method was employed as the primary analysis, estimating the weighted regression slope of the SNP-outcome effect on the SNP-exposure effect under the assumption of a zero intercept. To address potential pleiotropy and relax the assumptions of IVW, additional MR methods with varying model assumptions were utilized, including weighted median, Mendelian randomization robust adjusted profile score (MR-RAPS), MR-lasso, Robust IVW, Mendelian randomization pleiotropy residual sum and outlier (MR-PRESSO), and leave-one-out analysis (14). Each of these methods offers unique features that enhance their applicability. For example, MR-RAPS is particularly suited for addressing both systematic and idiosyncratic pleiotropy, making it a robust choice in scenarios with potential pleiotropic effects (15). Key features of MR-lasso and Robust IVW include penalizing the number of candidate SNPs and reducing the standard error of estimates, respectively.

To further assess the stability and reliability of the results, additional methods were employed, including penalized MR-Egger, robust MR-Egger, penalized robust MR-Egger, simple median, penalized weighted median, simple mode, weighted mode, penalized IVW, penalized Robust IVW, MR-constrained maximum likelihood (MR-cML), debiased inverse-variance weighted, and model-based estimation (MBE). The effect size was expressed as an odds ratio (OR) with a 95% confidence interval (CI) (16).

To address heterogeneity in the analysis, several methods were applied, including Cochran’s Q statistic, the I^2^ Index, and Rucker’s Q statistic (17). Funnel plots were used to visualize the data, while outlier SNPs were identified using MR-PRESSO and Cook’s distance (18, 19). Horizontal pleiotropy was assessed through intercept tests using the MR-Egger method (19). Additionally, the Phenoscanner database was utilized to identify and exclude pleiotropic SNPs that were directly associated with the outcome or confounding variables (20, 21).

Sensitivity analyses, including leave-one-out and single SNP MR approaches, were conducted to evaluate the robustness and reliability of the results (22). In our study, all statistical analyses were performed in R software 4.3.1 using 'TwoSampleMR', 'Mendelian Randomization', 'MRPRESSO', and 'mr.raps' packages (23, 24).

## 4. Results

**Figure 2.**
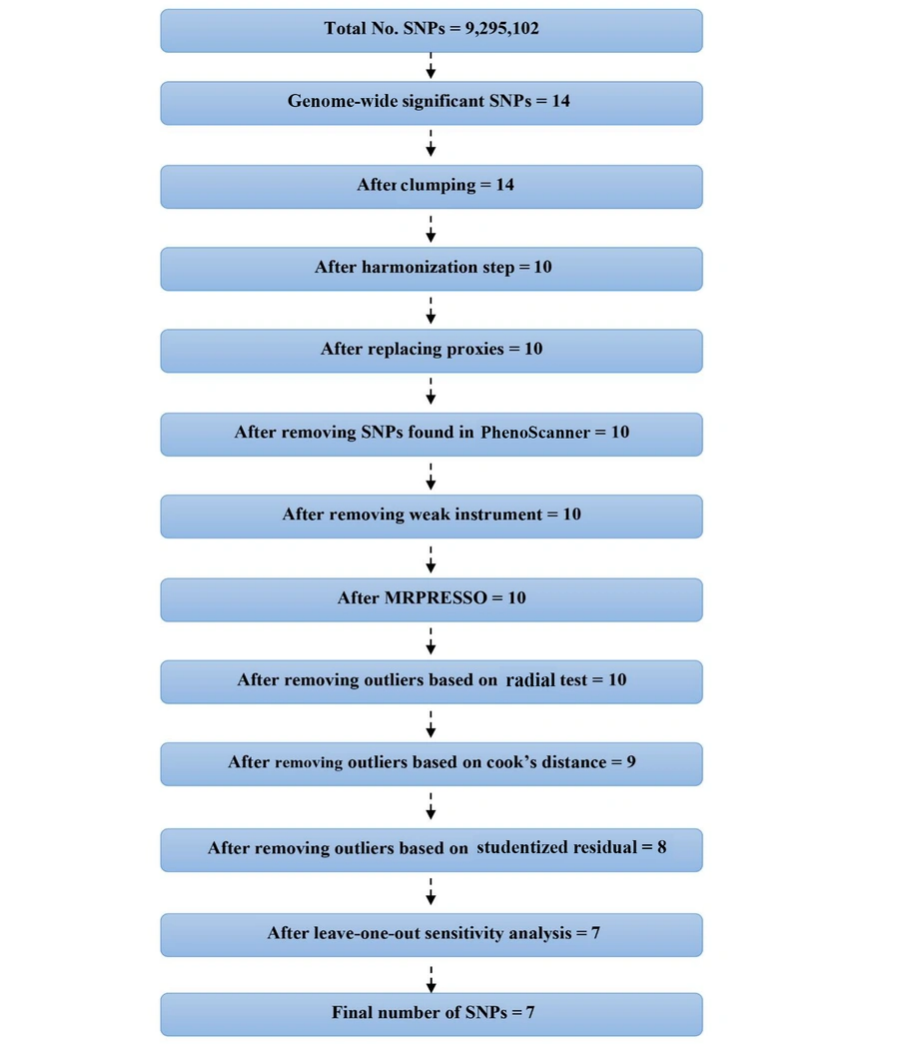
Flowchart for selection of genetic instruments for the MR analysis. Polycystic ovary syndrome (PCOS) was used as the exposure and Alzheimer’s disease (AD) as the outcome. Single nucleotide polymorphisms (SNPs) were filtered based on genome-wide significance, linkage disequilibrium (LD) clumping, allele frequency, and pleiotropy screening.

As shown in Figure 2, the initial GWAS for PCOS identified 14 SNPs that passed the genome-wide significance threshold (P < 5 × 10^-8^). Following clumping to ensure independence, 14 SNPs remained. Of these, four SNPs were identified as palindromic variants and were checked against the NIH LDproxy database for potential replacements using European (EUR) ancestry. However, no suitable replacements were found. After further screening for pleiotropy using PhenoScanner, 10 SNPs were retained for the MR analyses. The individual F-statistics for these SNPs ranged from 30.84 to 57.66 (Table 2), indicating sufficient instrument strength. The full details of the MR analyses and results can be accessed through the following HTML link: (https://akbarzadehms.github.io/PCOS-MR/).

The MR analyses showed no evidence of heterogeneity or horizontal pleiotropy, as indicated by the MR-Egger intercept P-value of 0.753 and the Q P-values of 0.272 and 0.207 for IVW and MR-Egger, respectively. The primary MR analysis using the IVW method found no significant association between genetically predicted PCOS and the risk of AD [OR = 0.967, 95% CI (0.905, 1.03); P = 0.311]. The weighted median, simple mode, and weighted mode methods yielded similar results.

**Figure 3.**
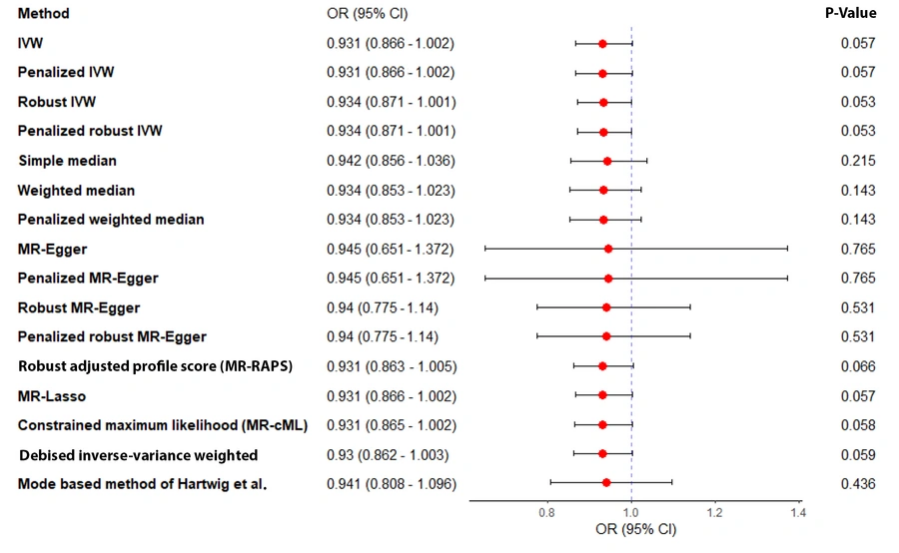
Estimated causal effects of polycystic ovary syndrome (PCOS) on Alzheimer’s disease (AD) using various Mendelian randomization (MR) methods. Odds ratios (OR) and 95% confidence intervals (CIs) are presented for each method.

**Figure 4.**
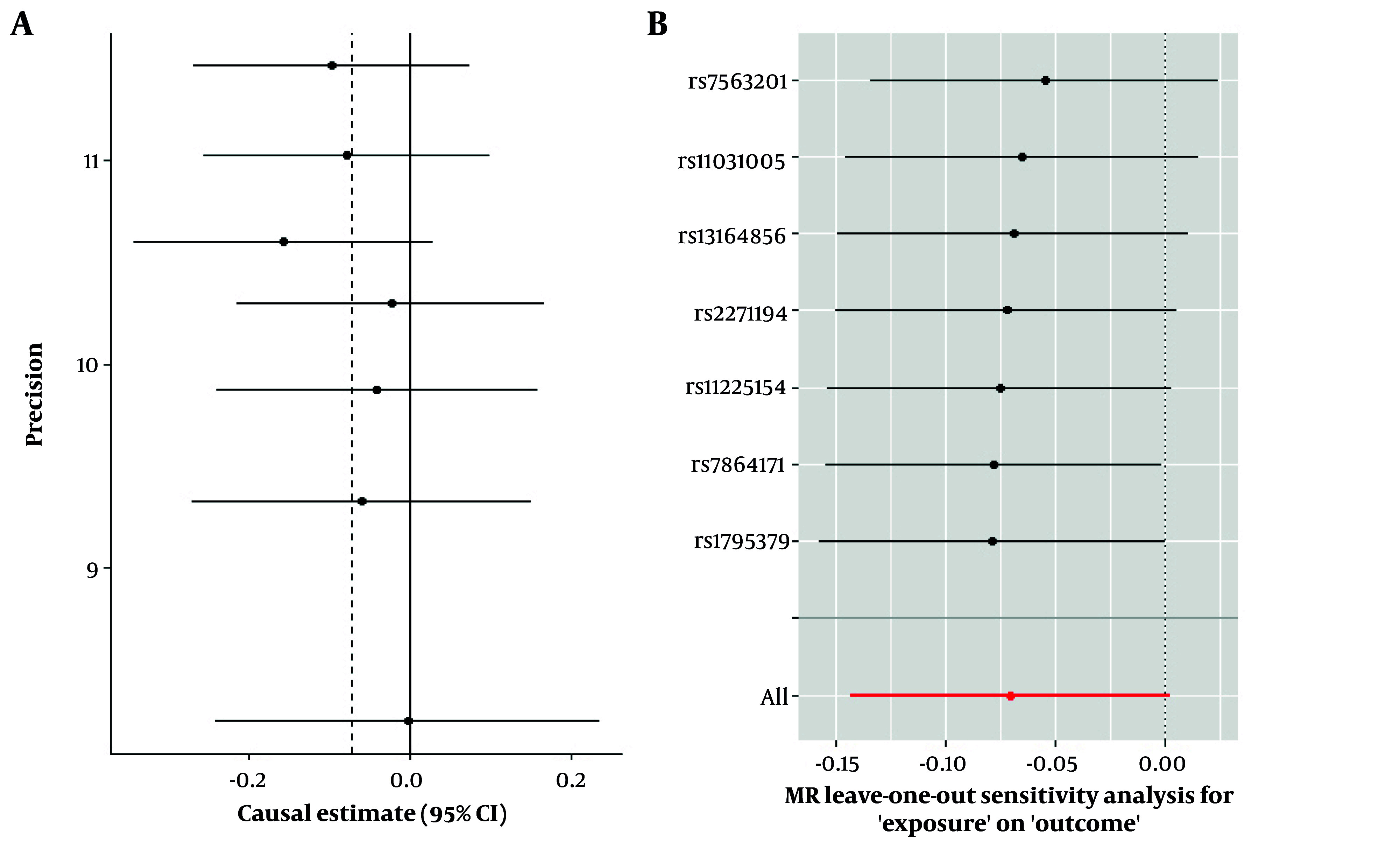
Diagnostic plots for assessing pleiotropy and robustness in MR analysis. A, funnel plot showing the distribution of individual SNP causal effect estimates; B, leave-one-out analysis indicating the influence of each SNP on the overall inverse variance weighted (IVW) estimate.

Sensitivity analyses, including leave-one-out, studentized residuals, and Cook’s distance, identified three SNPs (rs9696009, rs2178575, rs1784692) as potential outliers or influential observations. These SNPs were excluded from the final analysis. The final analysis with seven SNPs confirmed the absence of pleiotropy (MR-Egger intercept P-value = 0.943) and heterogeneity (Cochran’s Q = 1.57, P = 0.95). The IVW analysis again showed no significant association between PCOS and AD risk [OR = 0.93, 95% CI (0.866, 1.002); P = 0.057]. Other MR methods, including MR-Lasso, penalized IVW, MR-cML, and dIVW, provided consistent results (Figure 3). Funnel plots and leave-one-out sensitivity analyses further supported the robustness of these findings (Figure 4A and B).

## 5. Discussion

This study aimed to investigate the potential causal relationship between PCOS and AD using a two-sample MR approach. Our analysis did not identify a statistically significant causal effect of genetically predicted PCOS on the risk of AD. This finding suggests that, despite the shared clinical and metabolic features of these conditions, common genetic variants associated with PCOS do not appear to influence the development of AD. This reinforces the utility of MR in distinguishing correlation from causation by mitigating confounding and reverse causality, which often limit the interpretation of traditional observational studies.

Although our results do not support a direct genetic link, the complexity of both PCOS and AD invites further exploration. Numerous observational studies have reported associations between PCOS-related metabolic disturbances — such as insulin resistance, systemic inflammation, and hormonal imbalances — and cognitive dysfunction or dementia. For instance, insulin resistance, a hallmark of PCOS, has been implicated in impaired brain glucose metabolism and amyloid-β accumulation. Similarly, hormonal changes, such as elevated androgens or altered LH/FSH ratios, may influence neuroinflammatory pathways and synaptic plasticity. However, the absence of a genetic association in our MR analysis indicates that such mechanisms, if contributory, are likely driven by environmental or epigenetic factors rather than inherited genetic variation.

Mendelian randomization evaluates whether genetic variants associated with the exposure influence the outcome through the exposure. A positive association implies that altering the exposure (PCOS) would causally affect the outcome (AD), not that shared genetic variants directly link the two conditions. Mendelian randomization isolates the exposure’s effect from confounding environmental factors (e.g., diet, lifestyle) by leveraging genetic instruments. If MR shows no association (as in the PCOS-AD study), it suggests observed epidemiological links are likely driven by non-genetic mechanisms (e.g., environmental factors, epigenetic changes).

Several studies have explored this link, examining the intricate interplay of hormonal and metabolic factors in both conditions. For instance, a cohort study utilizing data from the CARDIA Women’s study demonstrated that women with PCOS exhibited poorer cognitive performance and reduced white matter integrity compared to those without PCOS, suggesting a possible association between PCOS and early brain health changes (25). Further investigations have revealed alterations in AD-related plasma proteins in women with PCOS, mirroring those observed in individuals with type 2 diabetes and strengthening the potential link between PCOS, T2D, and AD risk (26).

Several shared risk factors provide a biological basis for a potential connection between PCOS and AD. Hormonal imbalances, particularly an elevated LH/FSH ratio in PCOS, have been linked to Aβ accumulation and reduced BDNF in the brain, both of which can impact cognitive function. Metabolic disruptions, such as insulin resistance, a hallmark of PCOS, can impair hippocampal function, while free fatty acids contribute to inflammation and Aβ deposition, further connecting metabolic dysfunction to AD pathology. These findings are further corroborated by a retrospective cohort study, which reported a higher prevalence of dementia among women with PCOS compared to age-matched controls (7, 27).

However, it is crucial to recognize that PCOS may not only contribute to AD risk but also exert protective effects. Estrogen, a key hormone often dysregulated in PCOS, is well-recognized for its neuroprotective properties and its role in synaptic plasticity. Estrogen has been shown to inhibit beta-amyloid accumulation and activate genes associated with AD pathology, suggesting a potential protective effect against AD development (28, 29).

While the role of progesterone is complex, potentially both enhancing estrogen’s neuroprotective effects and counteracting its ability to prevent beta-amyloid buildup, androgens like testosterone can be converted to estrogen in the brain, potentially contributing to a neuroprotective environment. This intricate interplay of sex hormones and their effects on the brain may contribute to the lack of a clear causal link between PCOS and AD observed in our study (30-33).

Beyond the complexities introduced by sex hormones, other potential pathways might explain the relationship between PCOS and AD. Chronic hyperinsulinemia, a common feature in PCOS, can impair insulin signaling in the brain, leading to diminished glucose uptake, adversely affecting neuronal function, and promoting the production of amyloid-beta, a hallmark protein of AD (34). Additionally, the chronic, low-grade inflammation often observed in PCOS can trigger neuroinflammation, compromise the blood-brain barrier, and contribute to neuronal damage, potentially accelerating the progression of AD.

Hormonal imbalances in PCOS, particularly elevated androgens and LH, may exert neurotoxic effects, disrupt neurotransmitter systems, and impair synaptic plasticity, potentially contributing to cognitive decline. These findings collectively underscore the complex interplay of hormonal imbalances, metabolic disruptions, and inflammation in both PCOS and AD (7, 35, 36).

It is important to acknowledge that the impact of these factors on AD risk may be further modulated by the heterogeneity of PCOS and the presence of comorbidities. Women with PCOS who experience severe insulin resistance or obesity may be at an increased risk for AD compared to those with milder metabolic dysfunction. Furthermore, the presence of other comorbidities commonly associated with PCOS, such as cardiovascular disease or depression, may obscure the relationship between PCOS and AD (7, 8, 37-39).

Several studies have reported an elevated risk of AD in individuals with PCOS, particularly in postmenopausal women. This increased risk may be attributed to the disruption of the hypothalamic-pituitary-gonadal (HPG) axis. The HPG axis is essential for regulating reproductive hormones and is intricately linked to the hepatic biosynthesis of gonadal hormones, which can significantly influence brain health and susceptibility to neurodegenerative diseases (7, 40). In contrast, several studies have reported no significant association between PCOS and AD. This discrepancy may be attributed to several factors. The design of the studies themselves could significantly influence the outcomes; for instance, cross-sectional studies, which provide a snapshot in time, fail to capture the long-term effects of PCOS on cognitive function. Additionally, studies with smaller sample sizes may lack the statistical power necessary to detect subtle relationships between the two conditions. Variability in the assessment methods for cognitive function across studies can also contribute to inconsistencies; for example, self-reported cognitive decline may be less reliable than standardized neuropsychological tests (7, 25-27). Moreover, the specific phenotype of PCOS and the presence of comorbidities may significantly influence its impact on AD (7).

Our study adds to the growing body of literature suggesting that PCOS and AD may share common risk factors without necessarily sharing a causal genetic pathway. This underscores the need for integrative approaches combining genetic, epigenetic, environmental, and clinical data to fully understand the relationship between metabolic and neurodegenerative conditions. The primary strength of the present Mendelian study lies in its robust statistical approach. We employed advanced statistical techniques, including IVW, weighted median, and MR-Robust adjusted profile score (MR-RAPS), to verify the robustness of the results. Tests for pleiotropy and heterogeneity were conducted to evaluate the validity of the genetic instruments, which enhance the reliability of our findings by minimizing potential confounding factors.

Nonetheless, several limitations should be acknowledged. The GWAS data were predominantly derived from individuals of European ancestry, limiting the generalizability of our findings to other populations. Additionally, PCOS is a heterogeneous condition with multiple phenotypes that may have distinct underlying mechanisms and variable associations with AD. Our analysis did not stratify by PCOS phenotype or consider potential modifying factors such as obesity, comorbidities (e.g., depression or cardiovascular disease), or reproductive history. Lastly, MR can only assess lifetime genetic predisposition and cannot account for acquired or environmental influences that may mediate the PCOS-AD relationship. Future research may benefit from phenotype-specific genetic analyses, inclusion of diverse populations, and the integration of longitudinal, epigenetic, and metabolomic data. Such studies could help elucidate the non-genetic mechanisms that may underlie the epidemiological association between PCOS and AD.

### 5.1. Conclusions

This MR study found no evidence of a causal relationship between genetically predicted PCOS and the risk of AD. These findings suggest that shared genetic predisposition is unlikely to be a primary driver of the observed epidemiological associations between PCOS and AD reported in previous studies. While non-genetic factors may still play a role in this association, our results emphasize the importance of distinguishing correlation from causation and highlight the need for further research using alternative approaches to explore non-genetic mechanisms.

## Data Availability

The dataset presented in this study is available upon request from the corresponding author, either during submission or after publication. The data are not publicly available due to ethics concerns.
